# Serum protein biomarkers for juvenile dermatomyositis: a pilot study

**DOI:** 10.1186/s41927-020-00150-7

**Published:** 2020-10-01

**Authors:** Shefa M. Tawalbeh, Wilfredo Marin, Gabrielle A. Morgan, Utkarsh J. Dang, Yetrib Hathout, Lauren M. Pachman

**Affiliations:** 1 Biomedical Engineering Department, State University of New York at Binghamton, Binghamton, New York USA; 2 School of Pharmacy and Pharmaceutical Sciences, State University of New York at Binghamton, Binghamton, Johnson City, New York USA; 3 Department of Pediatrics, Northwestern’s Feinberg School of Medicine, Division of Pediatric Rheumatology, Ann and Robert H. Lurie Children’s Hospital; Cure JM Program of Excellence in Juvenile Myositis Research, Stanley Manne Children’s Research Institute of Chicago, Chicago, IL USA

**Keywords:** Juvenile dermatomyositis, SomaScan®, Serum proteomics, Biomarkers, Pharmacodynamic biomarkers

## Abstract

**Background:**

Blood accessible biomarkers to assess disease activity and their response to therapies in Juvenile Dermatomyositis (JDM) are urgently needed. This pilot study aims to identify serum protein biomarkers associated with clinical disease activity in untreated JDM and their response to medical therapy.

**Methods:**

SomaScan® technology screened JDM patients for 1305 proteins at three points: 1) before start of treatment, 2) while on therapy, and 3) after treatment tapering when patients were clinically inactive. To define disease associated biomarkers, SomaScan® data from untreated JDM patients (*n* = 8) were compared to SomaScan® data from an independent age-matched healthy control group (*n* = 12). Longitudinal analysis defined treatment responsive proteins at three time points: untreated (7 samples), treated (7 samples), and clinically inactive (6 samples). To confirm the SomaScan® data, a subset of nine candidate proteins (**CXCL11, IL-17B, IL-17D, IL-22, CXCL10, MCP-1, ANGPT2, MIF, IL-23) were** tested by ELISA after adding 2 JDM (one untreated, one clinically inactive) serum samples to the same group of JDM girls (8 untreated, 7 treated; 7 clinically inactive) as well as with 17 age, gender, matched healthy controls.

**Results:**

Comparison of untreated JDM versus healthy controls identified 202 elevated and 49 decreased serum proteins in JDM patients with an adjusted *p*-value < 0.001. Only 82 out of 251 identified biomarker candidates responded to treatment while 12 out of these 82 proteins returned to their original untreated disease levels upon therapy tapering. The ELISA testing of the untreated samples for nine candidate proteins confirmed previously known biomarkers (CXCL10 or IP-10, CXCL11 or I-TAC and MCP-1) and identified novel biomarkers including IL-22, Angiopoetin-2, and IL-17B in a cross-sectional analysis comparing 8 untreated JDM and 17 age/gender matched controls. The subsequent longitudinal data by ELISA were not concordant for some biomarkers (IL-22 and IL-17B), but the other biomarkers either normalized or rebounded concordantly.

**Conclusions:**

Blood accessible protein biomarkers reflecting JDM pathophysiology were identified; some of them rebounded after therapy was tapered. Further studies bridging these biomarkers to specific clinical features of JDM are required to confirm the clinical utility of these serum protein biomarkers.

## Background

Juvenile Dermatomyositis (JDM) is a complex, inflammatory, autoimmune disease targeting skeletal muscle, skin, and blood vessels [1]. JDM is also a rare disease, with an incidence of 3.2 cases/million children/year [2] that affects both genders, although the prevalence is higher in females (2.3:1) than in males [2]. The mean age of disease onset is 6.7 years and is often preceded by an infection [3]. Clinical features include diagnostic rashes often seen on the face, hands, and trunk, symmetrical proximal muscle weakness, and elevated serum levels of muscle derived enzymes [4]. Microvascular damage is displayed as capillary destruction/repair in the nailfold end row capillary loops [5], as well as in the inflamed muscle [6]. The duration of untreated disease impacts both laboratory data and clinical findings [7]; chronic inflammation is associated with the development of both lipodystrophy and dystrophic calcification in soft tissues [8, 9]. The disease course is very variable running from unicyclic to chronic continuous [10].

The tools currently available to assist medical personnel in assessing disease activity versus damage are limited in scope. Muscle function can be assessed by the Childhood Myositis Assessment Scale (CMAS) [11], but the range of *normal scores* achieved by healthy children *under the age of 4* at diagnosis, 26% of cases in our Registry, has not yet been established [12]. The Disease Activity Scores (Skin, Muscle and Total) [13] have been validated for use internationally [14]. In our experience, a child may appear to have quiescent JDM, but gene data, including RNASeq [15] and the SomaScan® data presented here, suggest continued activation of cytokine and metabolic systems among others (see below). Recent international collaboration has produced criteria for adults and children with dermatomyositis for both disease status and improvement [16, 17]; but, of note, both the parent and the physician global ratings are subjective.

The need for additional and more reliable outcome measures is crucial. Imaging techniques such as MRI, while more precise, remain costly and challenging to administer to young children, who often require sedation. An inexpensive, and reliable outcome measure would be most useful to guide administration of therapies in JDM that are currently largely empirical. These measures could be used to assess efficacy of a treatment, and to aid with go-no-go decision making in phase II clinical trials. In this context, blood accessible biomarkers are becoming attractive because they are less invasive, objectively measurable, inexpensive, and can be employed as surrogate outcome measures if validated. A set of blood accessible biomarkers, primarily those associated with muscle damage such as creatine kinase (CK), aldolase A (ALDOA), lactate dehydrogenase (LDH), alanine transaminase (ALT), and aspartate aminotransferase (AST) have been previously defined and are currently used in combination with other clinical outcomes to assess disease activity in JDM [18]. These biomarkers indicate muscle damage and are often influenced by exercise and, therefore, may not be specific to assess disease activity. It is well known that the standard serological biomarkers: CK, ALDOA, and AST/ALT may be elevated initially, but normalize within a 4–5 month period of untreated disease and that the duration of untreated disease impacts the JDM gene expression profile data as well as RNASeq data [7, 19]. In this pilot study, we sought to identify novel blood accessible biomarkers of JDM disease activity and evaluate their response to therapy.

## Methods

### Study participants

The CureJM Center of Excellence in Myositis Care and Research at The Ann & Robert H. Lurie Children’s Hospital of Chicago is one of four JM centers in the United States and has a designated JDM Registry and BioRepository (peripheral blood mononuclear cells (PBMCs), plasma, sera, nailfold capillary studies) every 6 months. This CureJM Registry has enrolled over 600 children with JM; currently, over 480 children have been diagnosed with JDM and their clinical and laboratory data is entered into a systematized data collection program (REDCap) for both new and return patients.

#### Children with JDM

For children with definite/probable JDM (defined by Bohan and Peter Criteria) [4], informed written consent was obtained from a parent/legally authorized representative (those under 18), along with informed written assent from adolescents aged 12–17.9, (Ann & Robert H. Lurie Children’s Hospital of Chicago, IRB# 2008–13,457, 2010–14,117). Well-characterized retrospective blood serum samples from 8 JDM female patients were stored at − 80 °C. All samples were de-identified, having a study code linking the clinical data to the sera, before they were accessed. These eight JDM patients were never treated before consenting to research sample collection, i.e., their first sample (*n* = 8; used for disease-associated biomarker investigations below) was drawn prior to any medical therapy. The second sample was obtained after treatment began and the third sample consisted of a time point at which patients’ treatments were being tapered off and they were clinically inactive (durations of time between samples is provided in Table 1). These patients sought treatment at The Ann and Robert H. Lurie Children’s CureJM Center of Excellence in Myositis Care and Research from March 2001, through April 2016. All samples were accessed from May 2017, through November 2018.

**Table 1:** Demographics and medications for JDM patients and healthy controls

	JDM^a^			Controls (SomaScan®)	Controls (ELISA)
	Baseline*n* = 8	On Treatmentn = 7	Inactiven = 8^b^	n = 12	n = 17
Age, yr, mean (stdv)	6.8 (2.7)	7.8 (2.7)	9.3 (1.9)	8.5 (1.7)	8.3 (2.3)
Gender					
Female, n	8	7	8	0	17
Male, n	0	0	0	12	0
DAS Total,^c^ mean (stdv)	11.0 (3.5)	4.6 (5.3)	0.6 (0.9)		
DAS Skin,^c^ mean (stdv)	5.6 (1.7)	1.9 (2.0)	0.3 (0.5)		
DAS Muscle,^c^ mean (stdv)	5.4 (3.3)	2.8 (3.4)	0.3 (0.6)		
Months between samples,mean (stdv)		7.4 (3.6)	20.8 (11.1)		
Drug, x = total patients on drug during study^d^	**Months on drug at sample** **mean (stdv)n = x**				
IV solumedrol, n = 7	*n* = 0	3.8 (1.0), *n* = 3	*n* = 0		
oral prednisone, n = 8	*n* = 0	6.6 (3.6), n = 7	20.8 (11.0), n = 3		
methotrexate, n = 7	n = 0	7.5 (3.9), *n* = 5	17.7 (0.9), *n* = 2		
cyclosporine, *n* = 2	n = 0	1.9, n = 1	n = 0		
mycophenolate mofetil, n = 5	n = 0	3.7 (3.1), n = 3	7.4 (6.8), n = 2		
hydroxychloroquine, *n* = 2	n = 0	4.9 (4.9), n = 2	27.2 (16.0), n = 2		

^a^ Same JDM patients were used for SomaScan® and ELISA assays except one missing datapoint for SomaScan® assay

^b^one patient sample not available for SomaScan®

^c^ ranges for DAS: Skin (0–9) + Muscle (0–11) Total DAS (0–20)

^d^one patient had received IVIG 2.7 years prior to the inactive sample

#### Disease activity scores (DAS)

It is our standard operating procedure to evaluate the children with myositis at each visit using this scoring system [13], which is validated and used internationally [20].

*Myositis Specific Antibody (MSA) and Myositis Associated Antibodies (MAA):* At the time of their first visit to our clinics, sera was obtained to determine these parameters, which utilized immunodiffusion and immunoprecipitation [2, 21]. The MSA of the tested patients were: three anti-p155/140, one anti-MJ, two had anti-p155/140 in combination with either positive or indeterminate Mi-2, one anti-MJ in combination with both an indeterminate anti-p155/140 and evidence of U5RNP that was determined to be too weak to confirm; one patient was negative for MSA.

#### Pediatric controls

In the first part of this pilot study, serum protein data for 12 healthy pediatric controls were from another independent study [22] and were employed as a bench mark for the initial exploratory comparison with the JDM data. In contrast, for the ELISA validation, healthy age, gender, matched controls were recruited (*n* = 17), and informed written consent was obtained from the parents/legally authorized representatives for those subjects aged up to 18 years, informed written assent obtained from those aged 12–17.9 years. (Ann & Robert H. Lurie Children’s Hospital of Chicago, IRB#2001–11,715) and, as for the children with JDM, their blood was drawn, centrifuged and processed with an arm to freezer time of 2 h or less, decreasing the likelihood of contamination of derived products from the formed blood elements.

### Serum proteome profiling using SomaScan® technology

Aliquots of 150 μl were prepared from all collected sera samples including untreated, while on treatment, and after start of treatment tapering (clinically inactive) time point samples and submitted for proteome profiling using high throughput multiplexing aptamer based SomaScan® assay targeting 1305 unique serum proteins (Somalogic Inc., Boulder, CO). The technique has been described in more details elsewhere [23, 24] and consists of a panel of protein-specific Slow Off-rate Modified DNA aptamers (SOMAmers) that bind with high specificity and affinity to target proteins. Each aptamer is tagged with a short DNA sequence enabling high throughput quantification using custom hybridization array. Protein quantities are recorded as relative fluorescent units (RFU) from microarrays. All arrays were done using a dilution series of each sera sample so that the signal/noise ratio of each aptamer/protein pair was optimized (three dilutions).

### MesoScale discovery® technique for biomarker validation

For data validation, we used serum samples from the same 8 female U-JDM patients prior to therapy (mean age 6.8 ± 2.7 years) as before, compared to 17 age, gender matched healthy female controls (mean age = 8.3 ± 2.3 years). We selected a subset of 9 candidate biomarkers based on the availability of a highly specific and highly accurate ELISA assay. Sera from all donors were assayed for C-X-C motif chemokine 11 (CXCL11 or I-TAC), Interleukin-17B (IL-17B), Interleukin-17D (IL-17D), Interleukin-22 (IL-22), C-X-C motif chemokine 10 (CXCL10 or IP-10), C-C motif chemokine 2 (CCL2 or MCP-1), Angiopoietin-2 (ANG-2 or ANGPT2), Macrophage migration inhibitory factor (MIF), and Interleukin-23 (IL-23) using the MesoScale® based ELISA assay. Dilutions for each assay were performed according to the manufacturer’s recommendation. For the U-Plex plates containing IL17B, IL17D, IL-22, CXCL10 (IP-10), CXCL11 (I-TAC), MCP-1, and TRAIL, the children’s sera was diluted 1:8. For a single spot U-plex plate for MIF, the sera was diluted 1:100. For V-plex plates to validate Angiopoietin-2, the sera were diluted 2-fold, while samples for IL-23 were diluted 16-fold. The plates were analyzed using MESO QuickPlex SQ 120 (Meso Scale Diagnostics, Rockville, MD). We also used a MesoScale**®** based ELISA assay to confirm the SomaScan® data for these nine candidate proteins in the longitudinal sera samples available for the seven JDM patients at three time points: 1) before treatment, 3) while on treatment, and 3) when clinically inactive and either off immunosuppression or tapering dosage.

### Data processing and statistical analysis

**Fig. 1:**
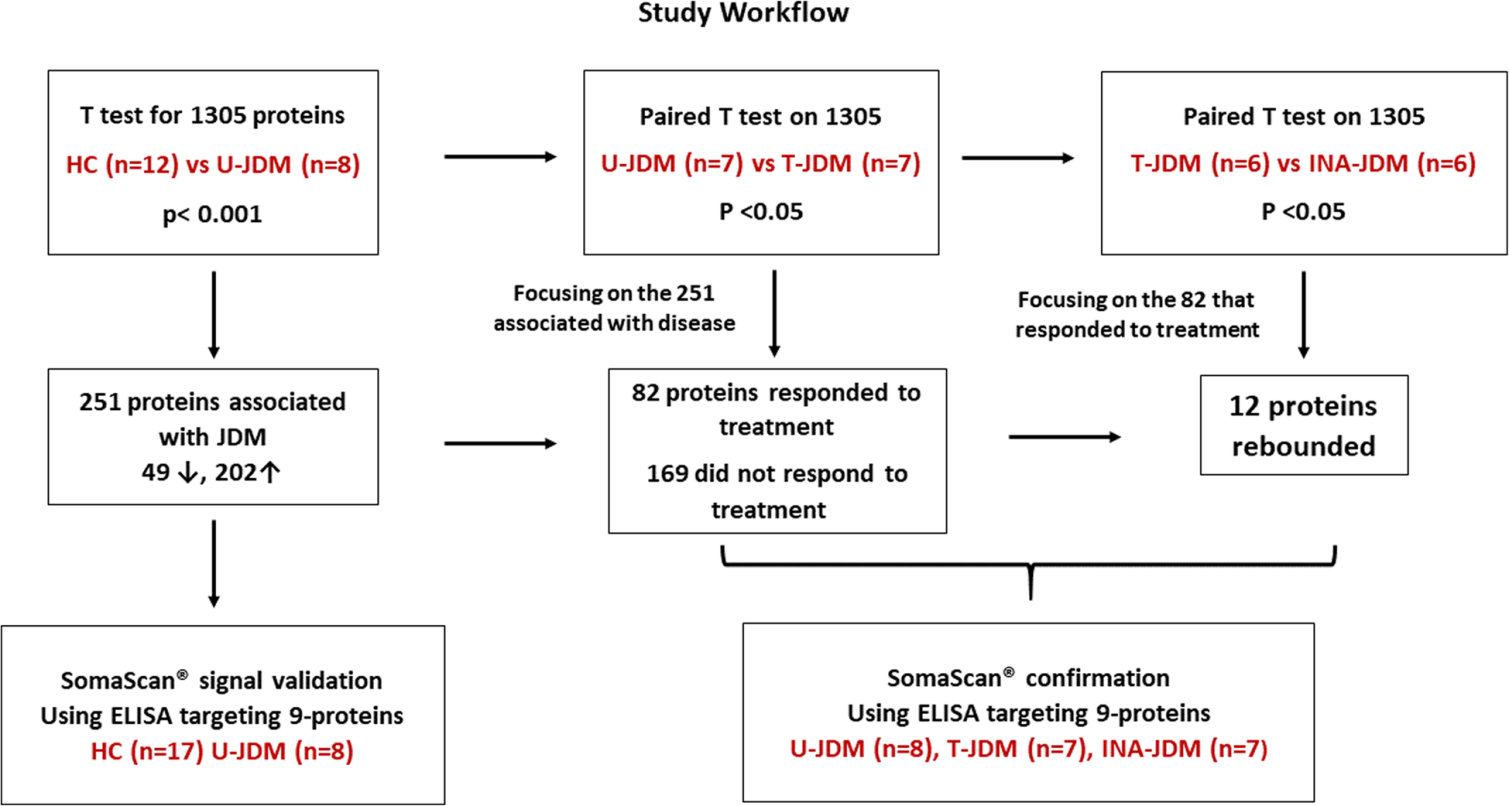
Schematic describing the study workflow. SomaScan® data (all samples at all time points were assayed simultaneously) while ELISA was used for testing concordance of signal for a subset of proteins. Abbreviations: Untreated JDM (U-JDM), on treatment (T-JDM), and clinically inactive JDM (INA-JDM)

Raw data generated from the SomaScan® analysis of all samples in this pilot study was first hybridization control normalized and median signal normalized, protein quantities were recorded as relative fluorescent unit (RFUs) then log2 transformed. As shown in the flow chart Fig. 1, we first performed a cross-sectional analysis to identify disease associated serum protein biomarkers significantly different in their levels between U-JDM (newly diagnosed, prior to any treatment, female, *n* = 8) and age-matched healthy controls (HC) (males, *n* = 12) in an initial exploratory step. All *p*-values were adjusted for multiple testing using a Benjamini & Hochberg false-discovery rate correction [25]. Subsequently, we performed two paired analyses: firstly, using 7 pre-treated (U-JDM) and on-treatment-JDM (T-JDM) and secondly, between 6 T-JDM and clinically inactive JDM (INA-JDM) samples (only 6 patients had sera for both data points available). Linear Models for MicroArrays and RNA-Seq Data (LIMMA) [26] was used for differential expression analysis for comparing 1305 serum protein biomarkers between U-JDM and matched controls, and for the paired analysis using samples from the three time points (U-JDM, T-JDM and INA-JDM). The empirical Bayes (moderated t-test) approach employed in LIMMA is particularly suitable for our proteomic data in that variation for protein data can be better estimated by reducing false positive findings and improving power. All statistical analyses were conducted using *R* [27]*.*

**Fig. 2:**
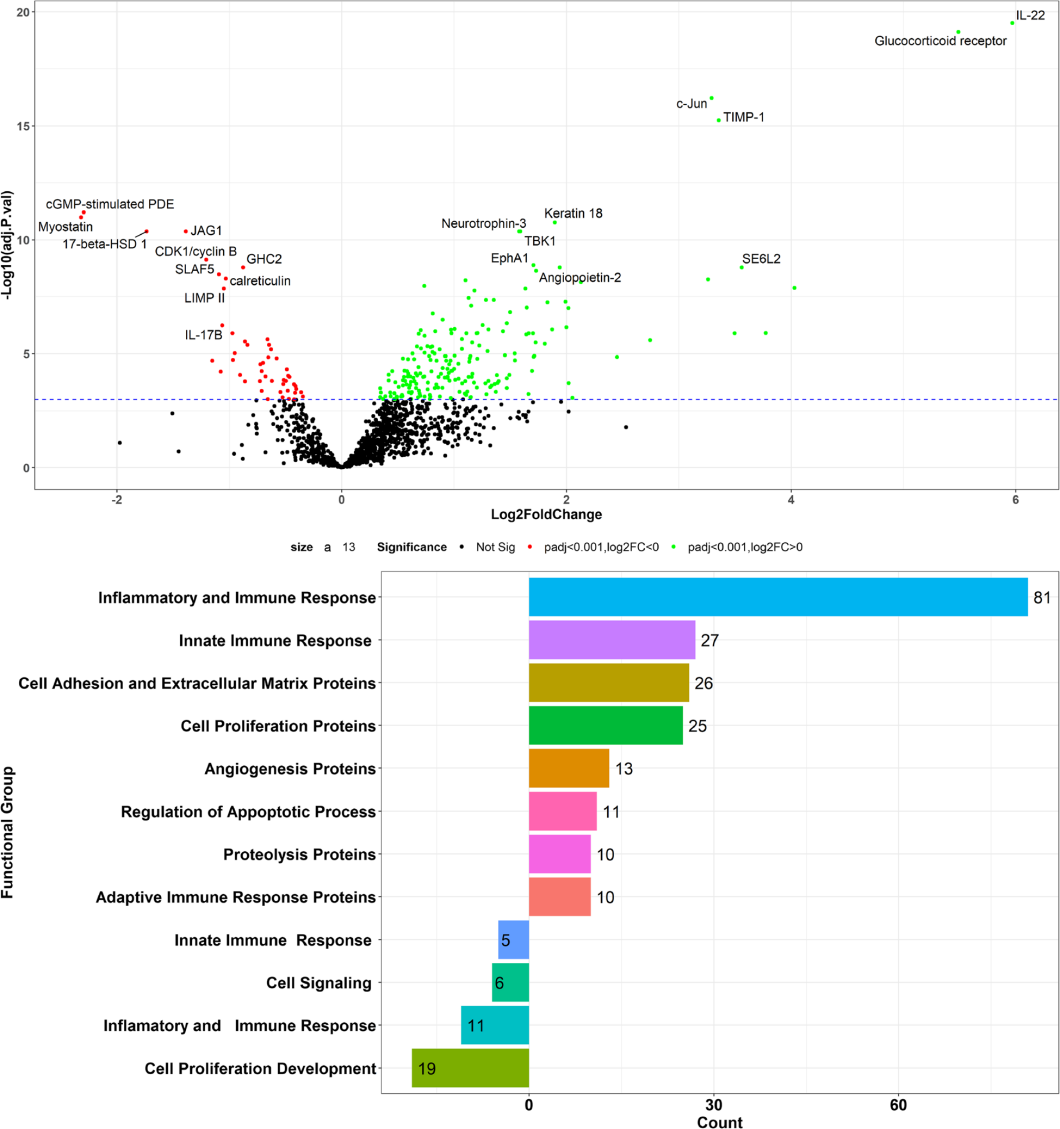
Comparison of SomaScan® serum proteome profiles of untreated JDM and healthy controls. The upper panel is a volcano plot showing significance versus log2 fold change in protein levels in U-JDM patients compared to healthy controls. SomaScan® RFU values were log2 transformed and plotted as log2 fold change for the 1305 proteins in U-JDM patients (*n* = 8) relative to healthy controls (*n* = 12). Green dots represent elevated proteins and red dots decreased proteins in sera samples of JDM patients relative to controls. The dashed blue line indicates the *p* value cutoff < 0.001 after adjustment for multiple testing. The most statistically significantly altered proteins are labeled from those decreased and increased in U-JDM vs HC are labeled. The lower panel shows a bar chart depicting the general biological function groups of some differentially altered proteins in blood of JDM patients relative to controls; the direction of the bars indicates whether this group was differentially increased (right) or decreased (left). Frequencies are provided within the bars

To group the identified JDM associated serum protein biomarkers according to their biological function, we used the Database for Annotation, Visualization and Integrated Discovery (DAVID) tool [28, 29]. The Uniprot accession IDs were uploaded to the functional annotation tool and “gene ontology” biological process was selected from the annotation summary result. Small groups consisting of less than 5 proteins were removed, and some manual curation was conducted using Uniprot [30], for example, grouping together immune and inflammatory proteins. Finally, a bar chart (Fig. 2 lower panel) was generated representing the biological function for groups of differentially altered proteins in blood of JDM patients relative to controls for both elevated and decreased proteins.

A cross sectional analysis was also performed on the ELISA data (8 U-JDM and 17.

age/gender HC) using LIMMA. This was followed by two paired analysis between U-JDM and T-JDM (*n* = 7) and between T-JDM and INA-JDM (n = 7). Pearson correlation was used to study the concordance of ELISA and SomaScan® data signals.

## Results

### Cross-sectional biomarker analysis in untreated JDM versus healthy controls

To identify biomarkers associated with JDM pathogenesis, we focused on U-JDM patients (*n* = 8). Cross-sectional comparisons identified 202 elevated and 49 decreased proteins (Supplemental Table S1; adjusted *p* values < 0.001) in U-JDM group (n = 8) relative to the healthy control group (*n* = 12). Since a variety of serum proteins differed in their levels between U-JDM patients and healthy controls, we focused on the most significant ones with adjusted p value < 0.001. The upper panel of Fig. 2 shows a volcano plot depicting the significance versus the log2 fold change of the 1305 proteins in U-JDM relative to healthy pediatric controls. Significantly elevated proteins and significantly decreased proteins in JDM relative to controls are represented by green and red dots, respectively.

**Fig. 3:**
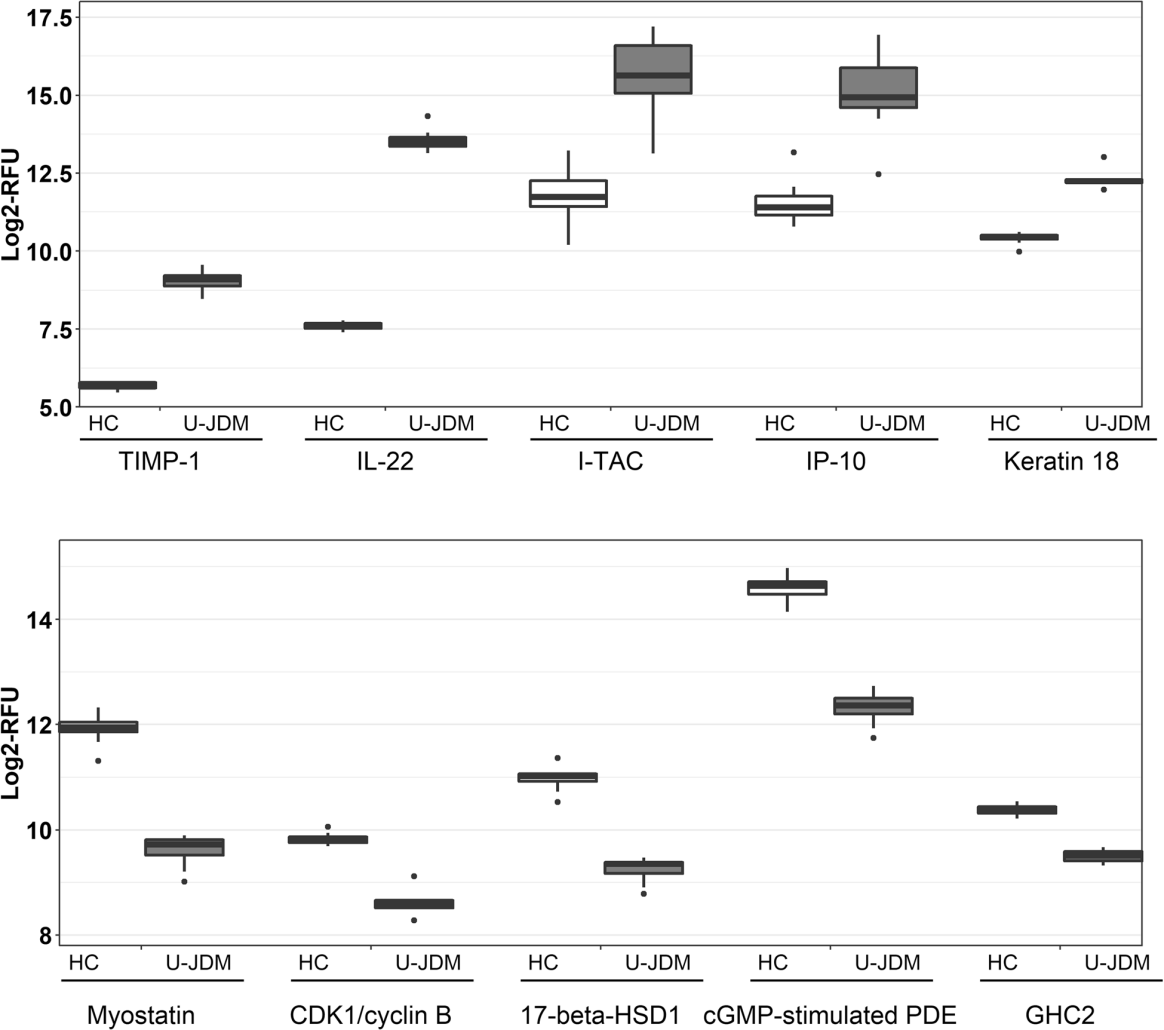
Boxplots of representative serum proteins that are significantly altered in their levels in U-JDM relative to controls. Top panel shows elevated proteins and bottom panel shows decreased proteins in sera samples of U-JDM patients (n = 8) relative to healthy controls (n = 12). *P* values for all comparisons are adjusted for multiple testing

As proof of principle, we identified previously reported JDM biomarkers including CXCL10 (IP-10), CXCL11 (I-TAC), MCP-1, MCP-2, MIP-1α, TNF-R1, MIG, IL-8 [18], as well as the classic muscle injury biomarkers for JDM: CK, aldolase A (ALDOA), Lactate dehydrogenase (LDH), ALT and AST. In addition to the previously known JDM biomarkers, we also identified several novel biomarkers (both elevated and decreased in U-JDM relative to controls) (Supplemental Table S1). Figure 3 shows examples of both known and novel serum protein biomarkers associated with JDM. Interleukin-22 (IL-22), tissue metalloproteinase inhibitor 1 (TIMP-1), and circulating keratin 18 are three novel biomarkers which were elevated by 62, 10, and 4-fold respectively in children with U-JDM relative to controls with an adjusted *p* value < 0.001. In contrast, myostatin, estradiol 17-beta-dehydrogenase 1 (17-beta-HSD 1) and cGMP-dependent 3′,5′-cyclic phosphodiesterase (cGMP-stimulated PDE) were decreased by 5, 3.33, and 5-fold, respectively in U-JDM relative to healthy controls with an adjusted p value < 0.001. A listing of JDM serum protein biomarkers, including those previously identified as well as novel JDM biomarkers, along with fold change and estimated significance values is presented in supplemental Table S1.

To classify the identified JDM associated serum protein biomarkers, we used the Database for Annotation, Visualization and Integrated Discovery (DAVID) tool [28, 29]. Figure 2 shows a bar chart grouping the biological function of differentially altered proteins in blood of U-JDM patients relative to controls. The *elevated* proteins were primarily associated with inflammatory and immune response, confirming previous studies [18] but also included a new group of proteins, such as those involved in innate immune response, e.g., complement component C6, complement C2, galectin-3, CD209 antigen, SLAM family member 6 (CD325 antigen), macrophage colony-stimulating factor 1 (M-CSF), lipopolysaccharide-binding protein and clusterin. Other significant proteins were those involved in angiogenesis and cell adhesion, e.g., **Angiopoietin-2 (**ANGPT2), vascular endothelial growth factor A (VEGFA), SHC-transforming protein 1 (SHC1) and Delta-like protein 4 (DLL4 laminin), vascular cell adhesion protein 1 (VCAM1), nidogen, coiled-coil domain-containing protein 80 (URB or CCD80), bone proteoglycan II (decorin), testican-2, integrin alpha-1:beta 1, von Willebrand factor.

The *decreased* serum circulating proteins in U-JDM, relative to controls, consisted primarily of proteins involved in cell proliferation, development and angiogenesis, e.g.,

myostatin (GDF8), growth/differentiation factor 9 (GDF9), pituitary adenylate cyclase-activating polypeptide (ADCYAP1), protein jagged-1(JAG1), fibroblast growth factor 18 (FGF18), gremlin-1 (GREM1), transforming growth factor beta-1 proprotein (TGF-b1), proheparin-binding EGF-like growth factor (HBEGF). Another group of *decreased* proteins were those involved in the immune, inflammatory, and innate immune response, e.g., interleukin-7 (IL-7), toll-like receptor 4 (TLR4), interleukin-17B (IL-17B), C-C motif chemokine 27 (CCL27) and tumor necrosis factor receptor superfamily member 10A (TRAIL-R1)). In addition, decreased proteins were involved in cell signaling, e.g., transforming growth factor beta-1(TGFB1), serine/threonine-protein kinase PLK1 (PLK1), activin receptor type-1B (ACV1B), tyrosine-protein kinase Lck (LCK) and mitogen-activated protein kinase 9 (MK09).

### Validation of a subset of candidate biomarkers by ELISA assay

To confirm the SomaScan® signal, a subset of significant protein biomarkers was selected for validation by ELISA assay. These included CXCL11 (I-TAC), IL-17B, IL-17D, IL-22, CXCL10 (IP-10), MCP-1, ANGPT2, IL-23, and MIF. These were selected based on the availability of a highly specific and sensitive ELISA assay. For this validation step, the same longitudinal sera samples collected at three time points were used: U-JDM (*n* = 7, mean age = 7.1 ± 2.7 yrs., all females); T-JDM (n = 7, mean age = 7.8 ± 2.7 yrs., all females); INA-JDM (n = 7, mean age = 9.5 ± 2.0 yrs., all females) and gender matched healthy controls (HC) (*n* = 17, mean age = 8.3 ± 2.3 yrs., all females). For the ELISA analysis, we added more controls to our analysis to increase our statistical power. The JDM samples were the *same* samples used in SomaScan® analysis. Table 2 shows the comparison between SomaScan® data and ELISA data on these 9 selected biomarkers along with fold change in untreated JDM relative to controls and respective adjusted *p*-values. Six of the 9 tested biomarkers were validated, and these include ANGPT2, CXCL11 (I-TAC), CXCL10 (IP-10), IL-17B, IL-22 and MCP-1.

**Table 2:** Biomarkers investigated with ELISA in sera samples of female U-JDM versus age matched female controls

8 **untreated** JDM vs 17 healthy control females						
Biomarker Name, (Accession Number)	SomaScan® assay		MSD ELISA assay		Confirm (Yes/No)	Previously reported by others or novel
	Fold Change	Adjusted P-value (direction)	Fold Change	Adjusted P-value		
**CXCL11** **(I-TAC)** **(O14625)**	13.7	< 0.001 (↑)	6.75	< 0.001 (↑)	Yes	[31]
**IL-17B (Q9UHF5)**	−2.1	< 0.001 (↓)	−2.4	< 0.001 (↓)	Yes	Novel
**IL-17D (Q8TAD2)**	−1.28	0.002 (↓)	−1.4	0.128 (↓)	No	[32]
**IL-22 (Q9GZX6)**	62	< 0.001(↑)	4.27	< 0.001 (↑)	Yes	Novel
**CXCL10 (IP-10)** **(P02778)**	11.3	< 0.001 (↑)	16.4	< 0.001 (↑)	Yes	[33]
**MCP-1 (P13500)**	2.1	< 0.001 (↑)	3.11	0.001 (↑)	Yes	[34]
**ANGPT2 (O15123)**	3.8	< 0.001 (↑)	2.6	< 0.001 (↑)	Yes	Novel
**MIF (P14174)**	2.38	< 0.001 (↑)	1.2	0.467 (↑)	No	[35]
**IL-23 (P29460, Q9NPF7)**	2.22	0.001 (↑)	−1.1	0.306 (↓)	No	Novel

C-X-C motif chemokine 11 (I-TAC or CXCL11), Interleukin-17B (IL-17B), Interleukin-17D (IL-17D), Interleukin-22 (IL-22), C-X-C motif chemokine 10 (IP-10 or CXCL10), C-C motif chemokine 2 (CCL2 or MCP-1), Angiopoietin-2 (ANG-2 or **ANGPT2),** Macrophage migration inhibitory factor (MIF) and Interleukin-23 (IL-23).

**Fig. 4:**
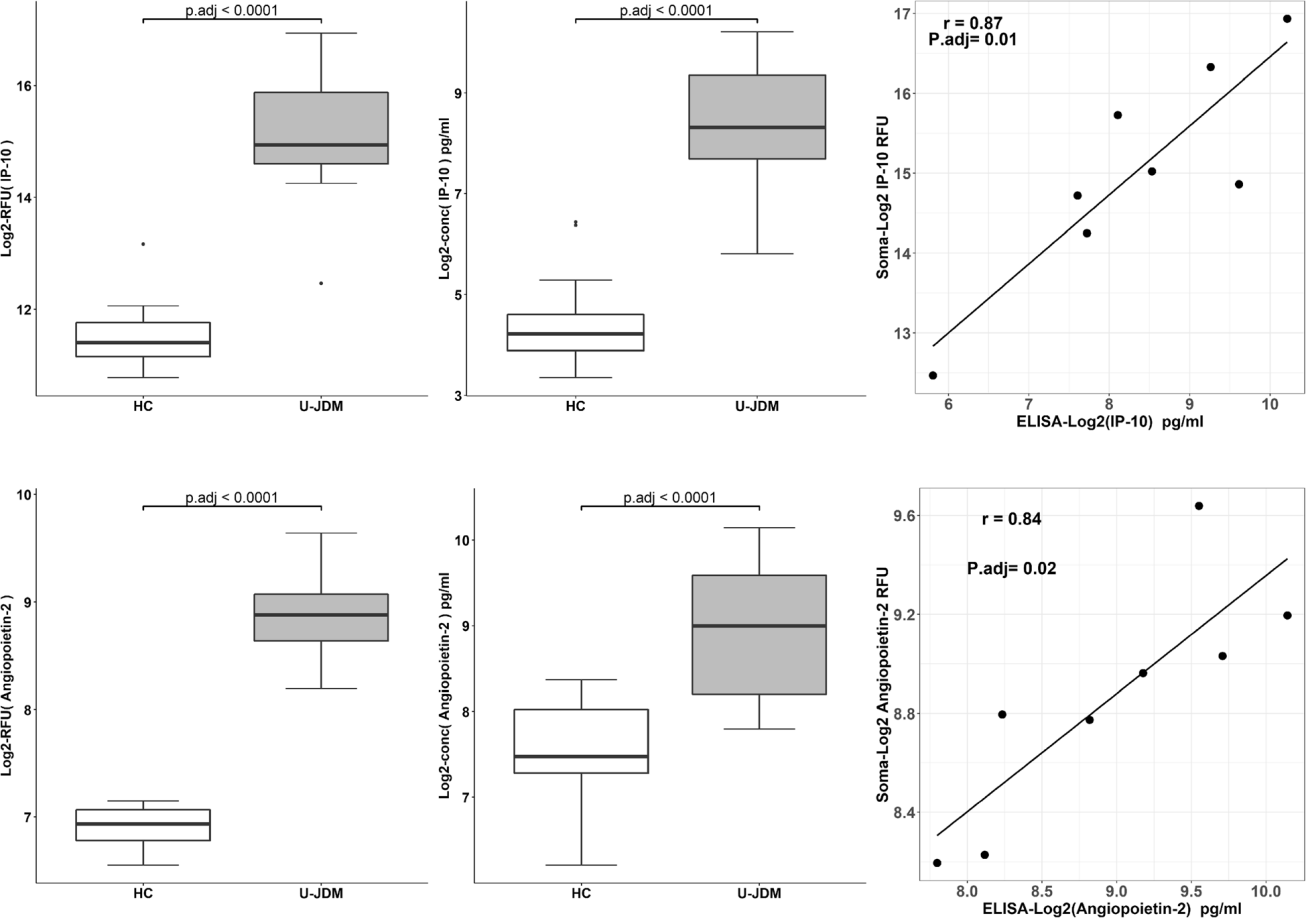
Correlation of SomaScan® data with ELISA data for two representative JDM biomarkers. Upper panel shows box plots for CXCL10 (IP-10) comparing healthy controls (n = 12 for SomaScan® and *n* = 17 for ELISA) to untreated JDM (U-JDM, n = 8) using SomaScan® data (left column) and ELISA (middle column) data with correlation plot on the right. Lower panel shows the same for **Angiopoietin**-2

CXCL11, CXCL10 and MCP-1 were recently reported by others [18], while IL-22, IL-17B and ANGPT2 are novel to this study. Figure 4 presents the strong correlation between SomaScan® data and ELISA assay for two exemplar biomarkers CXCL10 (IP-10) and ANGPT2 thus providing additional evidence for the validity of the SomaScan® data signal.

### Treatment responsive serum protein biomarkers in JDM - longitudinal analysis

**Fig. 5:**
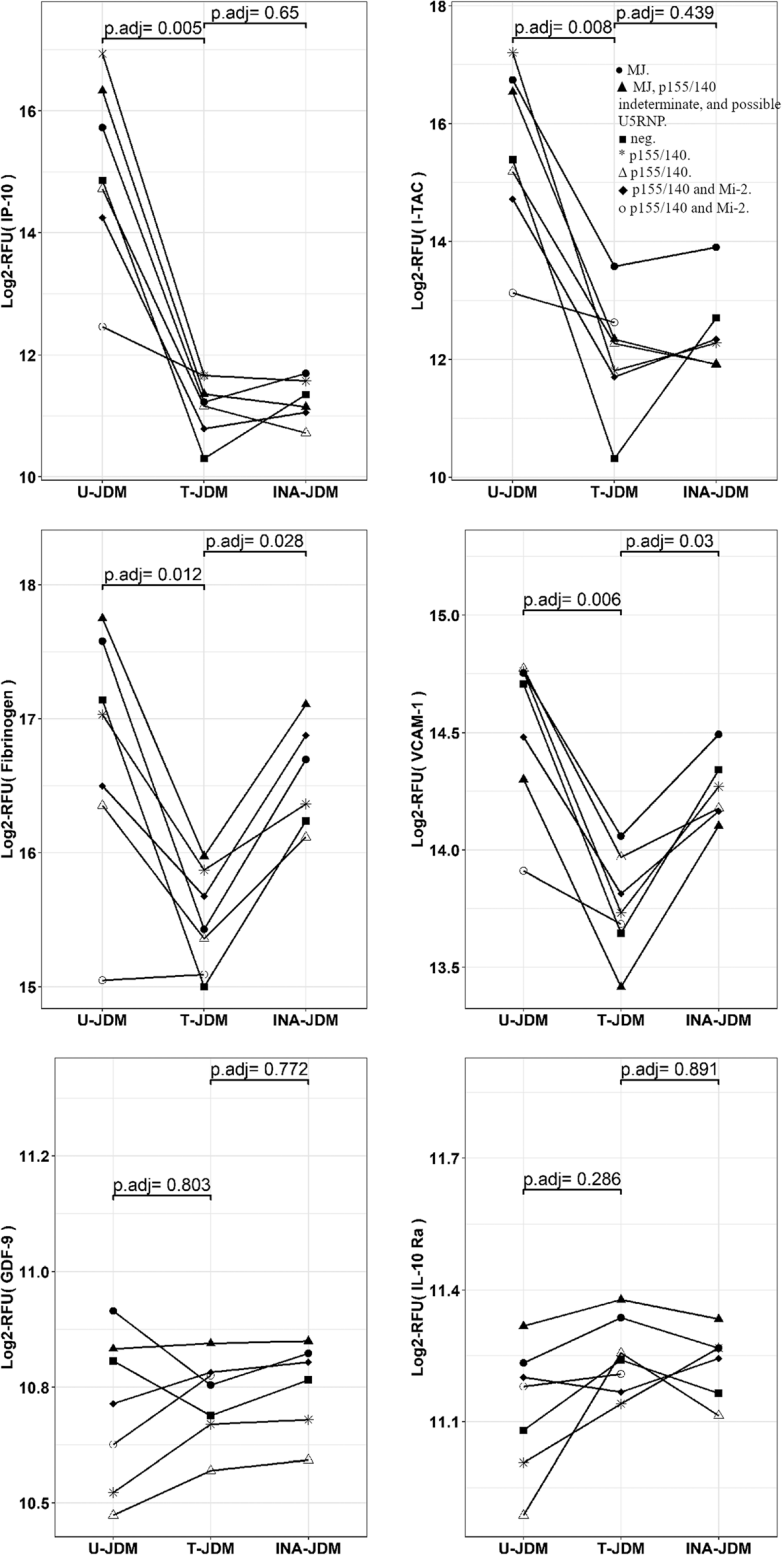
Examples of JDM serum protein biomarkers and their response to medical therapy. The upper panel shows biomarkers that normalized after treatment C-X-C motif chemokine 10 (IP-10) and C-X-C motif chemokine 11 (I-TAC). The middle panel shows fibrinogen and Vascular cell adhesion protein 1 (VCAM-1) decreased from untreated active levels before and while tapering therapy, when they appeared to be clinically inactive, and rebounded after tapering therapy. The lower panel shows biomarkers that did not respond to treatment: Growth/differentiation factor 9 (GDF-9) and Interleukin-10 receptor subunit alpha (IL10 -Ra). Symbols indicate the combination therapies for each patient: ● A, B, D. ▲ A, B, D, G. ■ A, B, E, F. * B, D. ∆ B, D, F.  ♦B, D, G.  οB, F. A = IV Solumedrol, B = oral prednisone; D = methotrexate; E = cyclosporine; F = mycophenolate mofetil; G = hydroxychloroquine. MSA profile for each patient is indicated in the insert in the figure

In this pilot study, 7 JDM had longitudinal samples collected at three time points: active disease before treatment, on treatment, and when treatment was being tapered off/clinically inactive patients (note that one patient did not have last time point for SomaScan® data). This allowed us to identify treatment responsive versus non-responsive biomarkers as well as biomarkers that rebounded back to pre-treatment levels after therapy tapering. As current treatment remains empirical, JDM patients received combination therapies in addition to prednisone, prescribed for all JDM patients (see Table 1 for detailed treatment regimen). Mean time from baseline sample to second sample was 7.4 ± 3.6 months while mean time from the “on treatment sample” to the “clinically inactive sample” was 20.8 ± 11.1 months. Of note, despite the varied combinations of Myositis Specific Antibody in this pilot study (see methods), the group as a whole behaved in a very similar fashion, except for the child who had a very faint line on MSA suggestive of overlap syndrome (Fig. 5).

Of the eight children in this pilot study, extended longitudinal data was available as follow-up data on six children. Of those 6 children, 4 had flares after the last sample date for this study. The mean time from last sample date to flare for the *n* = 4 was 15.8 ± 9.9 months. Two of these patients were off medications when their flares occurred and two were tapering medications. Two patients have yet to flare after the final sample. As for the remaining two patients, one patient’s last visit was the final sample date, while the other remained on a tapering schedule three years post-study but was then lost to follow up. Therefore, these preliminary data are suggestive that the increased levels of some serologically detectible proteins may be helpful to predict clinical reactivation of symptoms in some of the subjects studied but more in-depth investigations are needed.

Of the 1305 screened proteins, 272 proteins responded to treatment with an adjusted *p* value of < 0.05 but only 82 of these responsive biomarkers were associated with JDM at baseline while the remaining 169 disease-specific JDM biomarkers were not significantly impacted by treatment suggesting that this resistance might reflect unresolved disease activity despite extensive combination therapies. Interestingly, of the 82-treatment responsive JDM biomarkers, 72 remained normalized toward the levels seen in healthy controls after therapy tapering while 12 out of the 82 responsive biomarkers tended to return or rebound toward their original active disease state levels after therapy tapering. These include CD48 antigen (CD48), Cell surface glycoprotein CD200 receptor 1 (MO2R1), Coiled-coil domain-containing protein 80 (URB), Delta-like protein 1 (DLL1), Fibrinogen, Interleukin-23 (IL-23), Low affinity immunoglobulin epsilon Fc receptor (CD23), Sclerostin (SOST), Stem Cell Growth Factor-beta (SCGF-beta), Thrombospondin-2 (TSP2), Thrombospondin-4 (TSP4) and Vascular cell adhesion protein 1 (VCAM-1). Figure 5 shows the longitudinal trajectory of these different types of JDM biomarkers and their response to therapy. For example, CXCL10 (IP-10) and CXCL11 (I-TAC), known JDM biomarkers, normalized with treatment and stayed normalized even after therapy tapering, while fibrinogen and VCAM-1 normalized following treatment but rebounded during therapy tapering. Other biomarkers such as GDF-9 and IL10-Ra, although associated with JDM did not respond to treatment and remained unchanged in pre, post and after treatment tapering (see Fig. 5 lower panel).

Supplemental Figure S1 summarizes a bar plot depicting biological function grouping for biomarkers that responded to treatment. This included inflammation associated proteins, cell adhesion proteins and proteins associated with the innate immune response. These set of biomarkers might prove useful to assess response to therapy in JDM. The full name and function of these biomarkers is detailed in Supplemental Table S1.

**Fig. 6:**
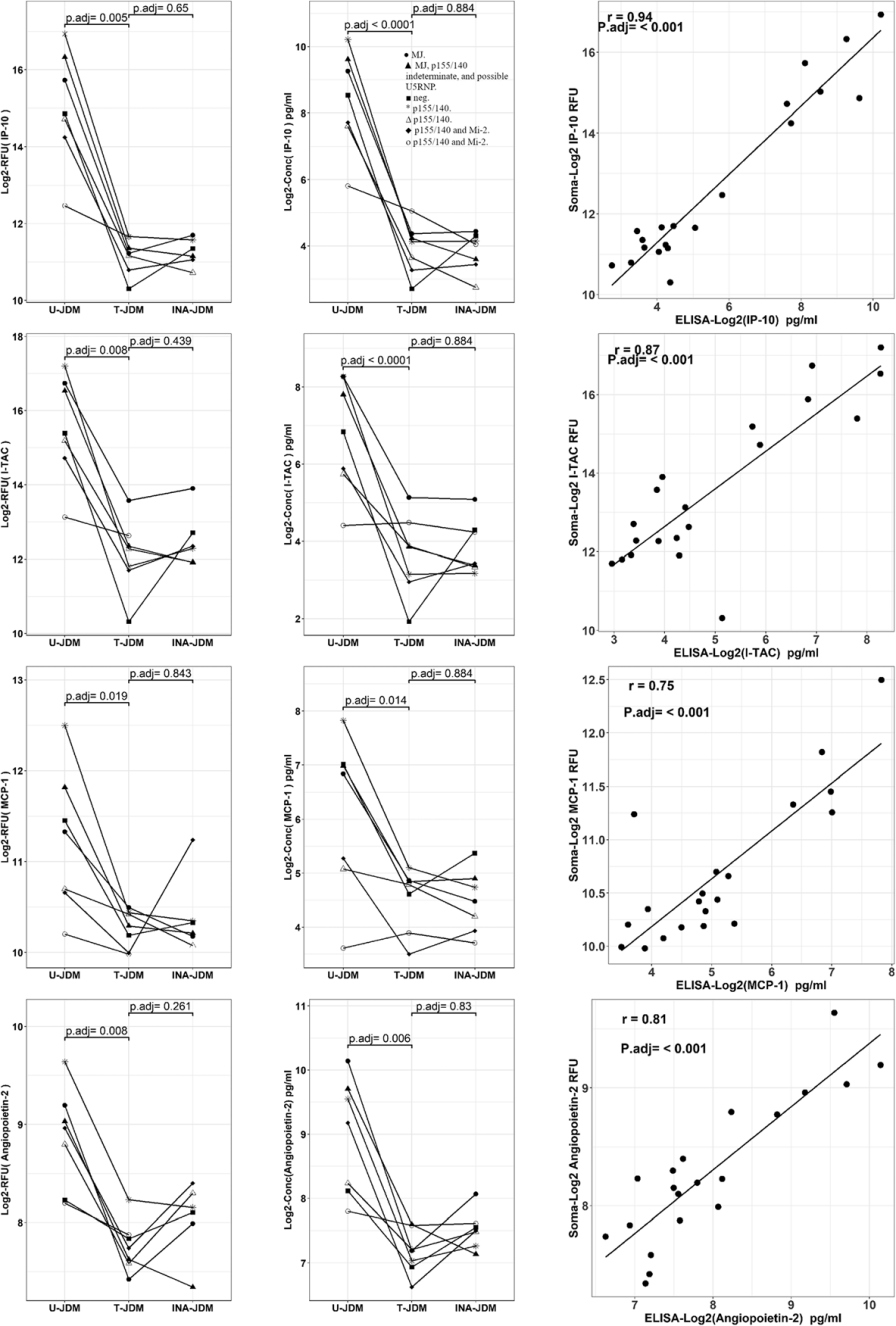
SomaScan® and ELISA data correlation for a subset of JDM biomarkers that responded to therapy. Left panel SomaScan® data (*n* = 7, 20 data points), Middle ELISA assay (n = 7, 21 data points) and right panel correlation graph. Symbols indicate the combination therapies for each patient: ● A, B, D. ▲ A, B, D, G. ■ A, B, E, F. * B, D. ∆ B, D, F. ♦B, D, G. οB, F. A = IV Solumedrol, B = oral prednisone; D = methotrexate; E = cyclosporine; F = mycophenolate mofetil; G = hydroxychloroquine. MSA profile for each patient is indicated in the insert in the figure

Response to therapy of the nine candidate proteins were further examined longitudinally using an ELISA assay. Figure 6 shows a subset of JDM biomarkers that confirmed the SomaScan® data (Fig. 6). Four out of the 9 tested candidate biomarkers (e.g. CXCL10, CXCL11, MCP-1 and ANGPT2) correlated well in their longitudinal trajectories between SomaScan® data and ELISA data with an r > 0.7 while the remaining 5 biomarkers (e.g. IL-22, IL-17B, IL-17D, MIF and IL-23) had a weaker correlation (− 0.44 < r < 0.11) for the longitudinal assays. Further experiments using orthogonal methods such as mass spectrometry are needed to resolve this discrepancy.

## Discussion

Using high throughput and highly multiplexing SomaScan® serum proteome profiling, we confirmed many serum protein biomarkers previously identified (e.g., CXCL10 (IP-10) and CXCL11 (I-TAC)) for the JDM population [36] but, importantly, identified several novel and potentially useful biomarkers (IL-22, Angiopoietin and IL-17B). In this pilot study, although it is a small cohort, we have identified an informative set of serum accessible protein biomarkers that are abnormal in children with JDM. The largest portion of the proteins differentially altered between controls and U-JDM were related to inflammation and the immune response, including Type 1 Interferon responsive biomarkers, well recognized as dominant components of the JDM inflammatory cascade [37]. Chemokines, cytokines, interleukins and soluble tumor necrosis factor receptors are the most common proteins listed in this group. Some of the biomarkers belonging to this class such as CXCL10 (IP-10), CXCL11 (I-TAC), MCP-1, Tumor necrosis factor receptor superfamily member 1A (TNF sR-I), C-C motif chemokine 21 (6Ckine), and tumor necrosis factor receptor superfamily member 21 (DR6), have been previously reported by others [18] but we have also identified additional biomarkers belonging to this group including IL-22, IL-23, IL-17B, C-X-C motif chemokine 13(BLC), IL-37, IL-34, macrophage colony-stimulating factor 1, osteopontin and many others (see supplemental Table S1). These biomarkers reflect the extensive systemic inflammation and auto-immune activation in children with JDM.

The second larger group consisted of proteins that are involved in angiogenesis and cellular development. For example, ANGPT2, VCAM-1 [38] and VEGF were significantly elevated in JDM relative to controls and regulate angiogenesis especially VEGF. Interestingly, while ANGPT2 and VCAM1 [38] responded to treatment and normalized, VEGF resisted response to treatment and remained elevated in JDM. This suggests unresolved disease activity despite combination therapies. This resistance of VEGF to therapy could be due to the fact that VEGF is increased in association with the profound myo-endothelial remodeling in JDM [39]. Also, of importance, given that JDM can often flare from a clinically quiescent state, these biomarkers that initially normalized with therapy and then reverted to “abnormal” as treatment is tapered, in the absence of detectible clinical activity, yet they may forecast a subsequent relapse of clinical disease [36]. That group included TSP-4, Il-23, VCAM-1 and CD48 and fibrinogen. Of note, CD48 binds to the natural killer cell ligand 284; natural killer cells have recently been more rigorously implicated in JDM tissue damage [40], while polymorphonuclear cells have also been recently identified [41].

Another significant class of JDM identified biomarkers are those associated with the innate immune system [42]. Circulating complement biomarkers included complements C2, C6 and C9, mannose-binding protein C, galectin-3, macrophage migration inhibitory factor (MIF), CD59 glycoprotein and many others (see supplemental Table S1). One mechanism for JDM is thought to involve activation of the classical complement pathway triggered by direct binding of C1q to injured endothelial cells [39]. A recent study confirmed the involvement of complement in muscle biopsies from JDM patients [43];further investigation is needed to establish the physiological significance of these circulating complement factors in JDM. Not only may complement be bound to circulating immune complexes, present in JDM plasma, [41], but the levels of C4 may be decreased as a consequence of decreased gene copy number [43].

In this study, we have also identified a novel class of serum protein biomarkers that were decreased in serum of U-JDM patients relative to controls. The majority of the decreased proteins consisted of developmental factors such myostatin (GDF8), GDF-9, GDF2, JAG-1, TGF-beta 1 as well as proteins associated with the innate immune system inflammatory response (see supplemental Table S1). Many of these decreased factors are involved in the adaptive immune response as well as angiogenesis. These findings complement a corresponding study of 30 untreated children with JDM and controls from the Cure JM Center of Excellence in Chicago, in which endothelial and inflammatory biomarkers—ICAM-1 and endoglin were associated with severe vasculopathy and loss of nailfold endrow capillary loops [31, 36]. In that study, elevated levels of galectin-9, CXCL10 and TNFR2 were predictive of a longer total treatment course [31, 36].

### Treatment effect on JDM biomarkers

Longitudinal analysis showed that only 82 proteins (32%) out of the 251 disease associated proteins responded to treatment. Of these 82 pharmacodynamic biomarkers, 72 biomarkers normalized after treatment (i.e., returned to healthy control levels after treatment) and 10 either decreased or increased further after treatment. It is important to note that 12 proteins returned to their active disease state after therapy tapering; these 12 biomarkers might be useful to predict flares, but further longitudinal sampling and analyses are needed to verify this hypothesis. Functional grouping showed that the proteins that normalized after treatment tapering were mainly related to immune and inflammatory response proteins followed by some cell adhesion and extracellular matrix proteins. The majority of proteins that did not respond to treatment were also associated with inflammatory and immune response despite the continued combination therapy with anti-inflammatory medications. To cite a few, IL-34, IL-16, IL-17, C-C motif chemokine 23, C-C motif chemokine 3-like 1, allograft inflammatory factor 1, CD70, and CD97, osteopontin are all associated with inflammation and immune response. They were significantly elevated in JDM and *did not decrease following treatment.* This suggests that there is a persistent state of inflammation in JDM patients even after treatment with several of our currently used anti-inflammatory agents. The second relevant group of JDM associated biomarkers that did not respond to therapy included those associated with innate immune (e.g., complement components C2, C6, and C9, macrophage migration inhibitory factor (MIF), mannose-binding protein C) as well as some angiogenesis regulators (e.g. VEGF, FGF18, Endothelial monocyte-activating polypeptide 2 and angiopoietin-4), suggesting that those pathways were still activated as well. Of note, the majority of the 12 biomarkers that rebounded after treatment tapering involved immune and inflammation related proteins (e.g. IL-23, CD23, Lymphotoxin a2/b1, CD48 antigen, fibrinogen and cell surface glycoprotein CD200 receptor 1), vasculature associated biomarkers (e.g. VCAM1 and TSP4) and bone formation regulating proteins (e.g. sclerostin and stem Cell Growth Factor-beta). This panel of biomarkers might prove useful to predict flares in JDM but further studies using a larger samples size and well controlled cohorts are needed to validate this observation. We anticipate that further examination of these treatment resistant biomarkers will bring insight into the complex nature of JDM pathophysiology and eventually lead to the development of novel therapeutic targets for JDM. It is clear that further studies are essential to define the physiological significance of these drug resistant biomarkers for children who have JDM.

Although preliminary, this pilot study had several strengths. The cohort used in this study to define disease associated biomarkers were never treated before, this was beneficial compared with other studies [44]. We also validated the SOMA data using a new cohort of 17 healthy female controls after the initial screening. Using longitudinal samples is useful because each patient can act as their own control in paired analysis. Furthermore, key JDM candidate biomarkers, both known (CXCL11 or I-TAC, MCP-1 and CXCL10 or IP-10) and novel ones (Angiopoetin-2, IL-22 and IL17B) were confirmed by ELISA assay for the untreated children with JDM further supporting the validity of this pilot data.

We acknowledge that small sample size is one important limitation of this pilot study. Although this small size sample was not adequately powered to draw a definite conclusion concerning the clinical utility of these biomarkers, we clearly identified a set of biomarkers that can be used in a panel to diagnose and assess disease activity and response to therapies in JDM. Of note, when we examined the subsequent clinical course of this small group of 8 children, 4 of the 8 children for whom further follow up data were available did subsequently flare, requiring increased immunosuppression. Further evaluation of the association of specific informative biomarkers with clinical outcomes is ongoing in our laboratory using a larger sample size to investigate the clinical utility of these biomarkers as surrogate outcomes to assess disease progression and response to therapies. We have yet to establish which of these biomarkers are indicative of disease severity, as well as responsive to potential treatment and can predict later outcomes.

Another limitation for the first phase of this pilot study is that healthy controls in SomaScan® data were not gender matched with the female JDM patients. Despite this fact, were able to confirm many of the previously reported biomarkers associated with JDM such as CXCL-10 (IP-10), CXCL-11 (I-TAC), MCP-1, MIF were abnormal, suggesting that there may be not be a major gender effect on identification of biomarkers associated with JDM. Moreover, we have followed the screening step by ELISA validation using a larger, age and gender matched healthy controls that confirmed our SomaScan® data. The gender effect was further examined by comparing data obtained on 3 males and 8 females for ANGP2 and there was no effect of sex on this new biomarker (data not shown). Nevertheless, future studies are planned using age and sex matched JDM and controls for both males and females.

## Conclusion

Although this is a pilot study of a limited number of children, the results represent the first large scale serum proteome profiling investigation that identifies a group of candidate serum protein biomarkers obtained from sera from children with well-defined definite/probable JDM. These children were tested at three stages of treatment: naïve, on therapy and after completion/tapering of medication when the children had achieved a clinical response, documented by the validated disease activity score. SomaScan® is a high throughput technology and enabled identification of a large number of biomarkers. A subset of SomaScan® identified biomarkers were confirmed by ELISA assay using age and gender matched healthy controls and JDM patients. In summary, this pilot study provides a comprehensive catalogue of JDM associated biomarkers that could be further tested and evaluated for their clinical utility using orthogonal techniques in the future, as well as designing the experiment with an appropriate sample size.

## Supplementary information

**Additional file 1 Table S1.** List of serum protein biomarkers that were altered in their levels in JDM patients relative to the heathy controls using SomaScan® technology, along with their response to treatment. For JDM associated biomarkers, data is presented as an average of Log2 transformed RFU values for each protein in untreated JDM (*n* = 8) and healthy controls (*n* = 12) and the fold change are given as Log2 fold change or raw RFU fold change JDM relative to controls. For longitudinal analysis of drug responsive biomarkers, data represents the average values for 7 pre and post treated JDM patients labeled as U-JDM (untreated JDM) and treated JDM. Biomarkers that responded to treatment are listed under column N as “normalized” while biomarkers that did not responded to treatment are listed as “Did not significantly respond”.

**Additional file 2 Figure S1.** Bar chart depicting the general biological function groups of some proteins that responded to treatment; the direction of the bars indicates whether this group was increased (right) or decreased (left) after treatment. Frequencies are provided within the bars.

## Data Availability

The datasets used and/or analyzed during the current study are available from the corresponding author on reasonable request.

## Abbreviations

JDM: Juvenile Dermatomyositis
CMAS: Childhood Myositis Assessment Scale
U-JDM: Untreated Juvenile Dermatomyositis
T-JDM: Treated Juvenile Dermatomyositis
INA-JDM: Clinically Inactive Juvenile Dermatomyositis
LIMMA: Linear Models for MicroArrays and RNA-Seq Data
DAVID: Database for Annotation, Visualization and Integrated Discovery
P.adj: Adjusted *P*-value
