# Supplementary figures and images for: Serum protein biomarkers for juvenile dermatomyositis: a pilot study
Source: BMC Rheumatol. 2020 Oct 1;4:52. doi: 10.1186/s41927-020-00150-7 (PMC7528471; doi:10.1186/s41927-020-00150-7)

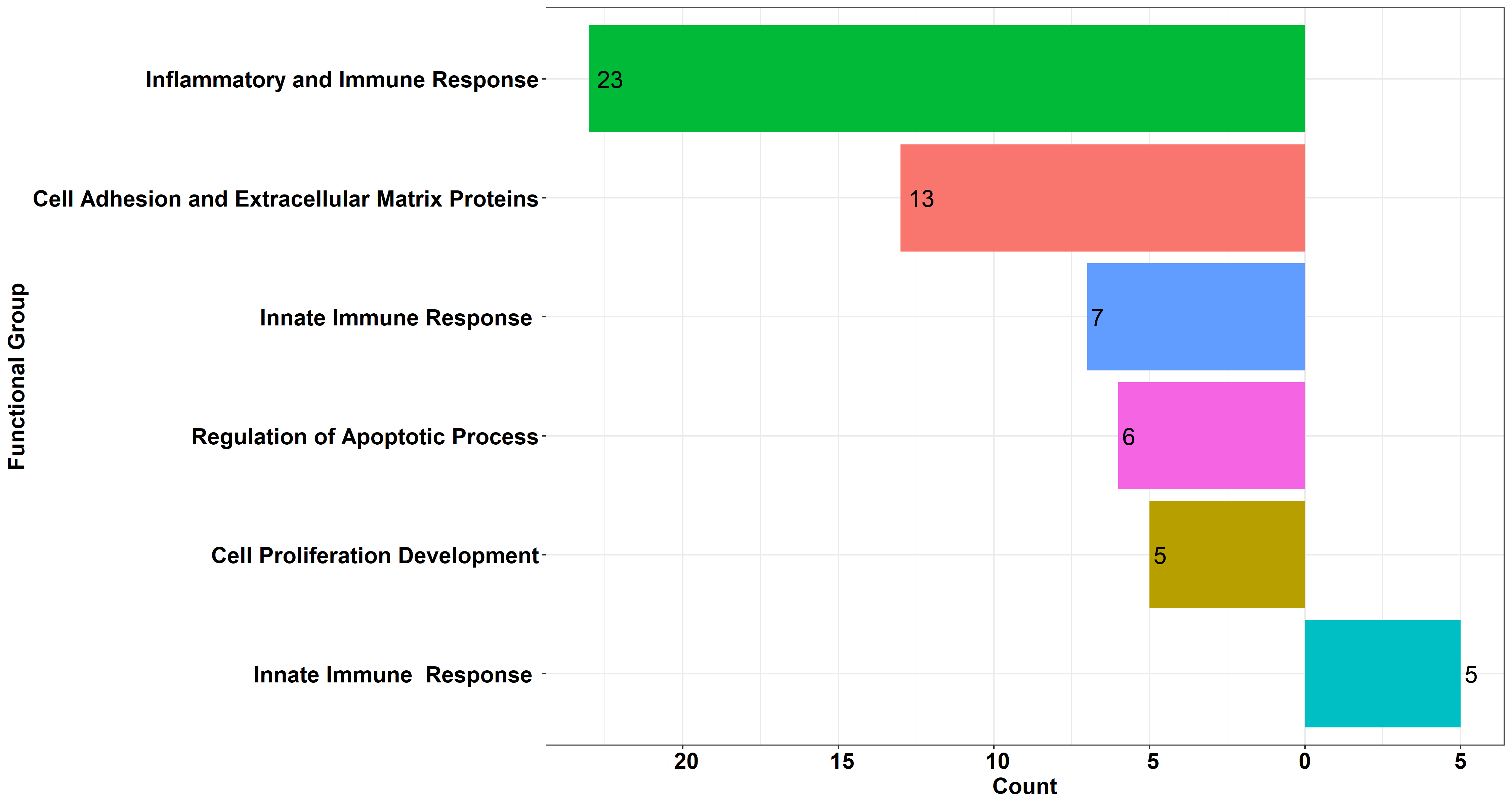

Supplement: Supplementary file 2 — Additional file 2 Figure S1. Bar chart depicting the general biological function groups of some proteins that responded to treatment; the direction of the bars indicates whether this group was increased (right) or decreased (left) after treatment. Frequencies are provided within the bars. [file 41927_2020_150_MOESM2_ESM.png]
